# Experiences with energy drink consumption among Norwegian adolescents

**DOI:** 10.1017/jns.2023.17

**Published:** 2023-02-23

**Authors:** Siri Kaldenbach, Tor Arne Strand, Mads Nicolaj Holten-Andersen

**Affiliations:** 1 Sykehuset Innlandet HF, Department of Paediatric and Adolescent Medicine, Innlandet Hospital Trust, Lillehammer, Norway; 2 University of Oslo, Institute of Clinical Medicine, Oslo, Norway; 3 Sykehuset Innlandet HF, Research Department, Innlandet Hospital Trust, Lillehammer, Norway; 4 University of Bergen, Centre for International Health, Bergen, Norway

**Keywords:** Adolescents, Energy drinks, Population-based, Public health

## Abstract

The objective of the present study was to describe adolescents’ habits and experiences with energy drink (ED) consumption and the relation to the amount of ED consumed. We used the national cross-sectional study Ungdata, conducted in 2015–16 in Norway. A total of 15 913 adolescents aged 13–19 years answered questions about ED consumption related to the following topics: reasons for, experiences with, habits and parental attitudes. The sample comprised only adolescents reporting to be ED consumers. We estimated the association between the responses and the average daily consumption of ED in multiple regression models. Those who consumed ED ‘to concentrate’ or ‘to perform better in school’ consumed on average 73⋅1 (CI 65⋅8, 80⋅3) and 112⋅0 (CI 102⋅7, 121⋅2) ml more daily, respectively, than those who did not consume ED for these reasons. Up to 80 % of the adolescents reported that ‘my parents think it is OK that I drink energy drink’, but at the same time almost 50 % reported that ‘my parents say that I shouldn't drink energy drink’. Apart from increased endurance and feeling stronger, both desired and adverse effects of ED consumption were reported. Our findings indicate that the expectation created by the ED companies have great influence on the adolescents’ consumption rate and that parental attitudes towards ED have little to no influence on the adolescents’ consumption rate.

## Introduction

Energy drinks (ED) are popular beverages containing high levels of caffeine, sugar and additional ingredients with stimulating properties^(1)^. ED are generally considered an energy-dense and nutrient-poor beverage^(2)^ and are usually advertised to give an extra boost in energy, mood, physical and cognitive performance. Examples of popular ED brands sold in Norway are Red Bull, Battery, Burn and Coca-Cola Energy to mention some^(3)^. ED have become a central part of partying and sport culture, especially among adolescent boys who report that they use ED to enhance their sports performance^(4,5)^. In Norway, the sales of ED have increased markedly in recent years with more than 62 million litres sold in 2021 only^(6)^.

Adolescents are exposed to marketing of ED through different channels such as online gaming, TV, social media, advertisements of sporting events and sponsorship contract (e.g. athletes, football teams)^(7–9)^. According to the cross-sectional study by Buchanan *et al.*, a small group of young adults (*n* 359) who consumed ED were significantly more exposed to digital marketing of ED, than the non-ED consumers^(10)^. In the same study, only engagement with digital marketing, when comparing to exposure and engagement of other types of marketing, significantly increased the likelihood of consuming ED when controlling for other covariates (age, sex, socio-economic status, student and work status, perceived significant others’ acceptance of their ED use). Digital marketing of ED has proven to be effective in persuading young adults to purchase and consume ED^(11)^. In addition, online marketing could have a greater impact on young adults who are less aware of the possible negative health outcomes of consuming ED^(11)^. Lately, digital marketing through social media has been linked to unhealthy food consumption among children^(12,13)^, as exposed children increased their intake of calories and unhealthy snacks^(14)^.

Boys have been shown to consume larger amounts of ED than girls, which could be due to the masculinity of advertisements and marketing of ED and how potential effects of ED consumption include better physical and sports performance^(7,15)^. In previous studies, we found that the typical ED consumer was indeed a boy who had a lower socio-economic status and had spent much of his free time on screen-related activities^(16,17)^. This is in line with other studies which have found positive associations between sedentary behaviour and screen time activities^(18,19)^. Yet positive associations between increased physical activity and ED consumption has also been found, which might suggest the use of ED as a refreshment or even a booster during sport activities^(17,20)^.

Despite the positive marketing of ED, several recent studies have found potential negative effects of ED consumption. ED consumption has been found to be associated with heart palpitations^(21)^, higher systolic blood pressure and increased QT interval (the time it takes for the ventricles of the heart to contract and fully relax)^(22,23)^. In addition, increased headache and sleeping problems have also been linked to ED consumption ranging from a single high dose (946 ml ED with 320 mg caffeine) to daily use^(20,24)^. These negative effects are most likely related to the high caffeine content of ED, though the combination of ingredients found in ED might also play a role. According to a review by the Norwegian Scientific Committee for Food and Environment, an adolescent with an average weight of 50 kg would be at risk of sleep disturbance when consuming more than 70 mg/d of caffeine, which is approximately 500 ml of ED a day^(1,25)^.

At the moment, there are no regulations or legislations in Norway limiting adolescents from consuming ED^(1)^. Social circumstances and parental attitudes towards ED consumption, either disapproving or encouraging consumption, have shown to affect ED consumption^(5)^. Yet, broader knowledge on attitudes, habits and experience with ED consumption among adolescents is scarce. The aim of the present study was to describe the associations between experiences, attitudes and expectations of ED consumption related to the amount of ED consumed in the adolescent population.

## Experimental methods

### Study design and participants

Data from the Norwegian annual Ungdata survey were used for the study. Ungdata is a national youth survey aiming to survey adolescent health and is typically performed every third year in each of the Norwegian municipalities^(17)^. The survey is administered by the Norwegian Social Research (NOVA) at Oslo Metropolitan University in a collaboration with the Regional Drug and Alcohol Competence Centres (KoRus). An electronic questionnaire is filled out during school hours by students in lower and upper secondary school (Grades 8–10 including ages 12–15 years and grades 11–13 including ages 15–19, respectively). Details regarding the data are available at https://ungdata.no/english/^(26)^.

The data used in the present study were collected in 2015 and 2016 and encompassed 15 913 adolescents between the ages of 13 and 19 years (57⋅9 % male, 38⋅8 % female). For the purposes of the current analysis, we chose to only include those who reported consuming ED.

### Measures

The main outcome variable was average daily ED consumption. This was estimated from two questions: ‘How often do you drink energy drinks?’ with options on a seven-point scale from ‘never’ to ‘daily’, and ‘How much energy drink do you usually drink when you consume energy drinks?’ with options on a six-point scale from ‘one small can (ca 250 ml)’ to ‘several cans corresponding to more than 1⋅5 litres’. Respondents were defined as ED consumers if they answered the question ‘How often do you drink energy drinks?’ with the answer options ‘Once a month or less’ up to ‘daily’.

Adolescents were asked about their ED consumption regarding: (1) reasons for drinking ED; (2) experiences with ED consumption; (3) parental attitudes towards ED consumption and (4) circumstances when ED are usually consumed. Each statement could be answered with one of four incremental options from ‘not true’ to ‘very true’. The responses were dichotomised from four into two categories on all four topics: ‘disagree’ and ‘agree’.

Reasons for drinking ED were assessed by the following questions and statements: The question ‘To what extent do the following statements describe why you drink energy drinks?’ was followed by seven statements: ‘Because it tastes good’, ‘Because I need energy’, ‘Because I need to stay awake’, ‘Because my friends do it’, ‘To perform better in sports’, ‘To concentrate’, ‘To perform better in school’. Experiences with ED consumption were assessed using the question ‘How often do you experience any of the following after drinking energy drinks?’ followed by eight statements: ‘increased endurance/less tired’, ‘feel stronger/have extra energy’, ‘feel nervous’, ‘have difficulty sleeping’, ‘do not feel anything in particular when I drink energy drinks’.

Parental attitudes were assessed using the question ‘How well do the following statements fit regarding your parents’ attitudes towards energy drinks?’ followed by four statements: ‘my parents think it is OK that I drink energy drinks’, ‘my parents don't know that I drink energy drinks’, ‘my parents say that I shouldn't drink energy drinks’, ‘my parents have spoken to my about energy drinks’.

Circumstances when ED are usually consumed was assessed using the question ‘What do you usually do when you drink energy drinks?’ followed by eight statements: ‘I am with friends’, ‘I am at a party’, ‘I am on my way to/doing sports’, ‘I am at the youth club’, ‘I am on my way to or from school’, ‘I am gaming/using my computer’, ‘I am watching TV, movies or streaming’, ‘I am at home’.

All participants reported on sex (male, female), grade (8–13), in addition to the other background variables including leisure screen time, perceived family economy, centrality and physical activity. Leisure screen time was assessed using the question ‘Outside school, how much time do you normally spend on activities that involve looking at a screen (TV, computer, tablet, mobile phone) each day?’ with response options on a seven-point incremental scale ranging from ‘no time’ to ‘≥ 6 h’. The first three options of the variable leisure screen time were merged into one response of ‘≤2 h’ due to the relatively low number of respondents in each of these categories. Physical activity was assessed by the question ‘How often do you perform physical activity which gets you out of breath or makes you sweaty?’ with response options on a six-point incremental scale ranging from ‘never’ to ‘at least five times a week’. Perceived family economy was based on the question ‘has your family's economic situation been good or bad during the past two years?’, with five response options ranging from ‘always good’ to ‘always bad’. Finally, the Norwegian centrality index was used to define centrality which refers to an index of travel time to workplaces and service functions from all populated basic units. Group 1 contains the most central municipalities (highest index) and group 6 contains the least central (lowest index)^(27)^.

### Statistical analyses

We used linear regression models to estimate the crude and adjusted mean differences in ED intake in ml/d between the response categories from the different questions in the four categories: (1) reasons for drinking ED; (2) experiences with ED consumption; (3) parental attitudes towards ED consumption and (4) circumstances when ED is usually consumed. Responses to the different questions are presented with percentages while the regression coefficients are additionally presented with 95 % CIs. All estimates were adjusted for gender, grade (as a proxy for age), leisure screen time, perceived family economy, centrality and physical activity, but these estimates, which were chosen based on previous knowledge^(16,28)^, are not presented and were chosen *a priori*. The plot describing the association between the average daily intake of ED and the frequency of ED was generated using two-way fractional polynomials (‘twoway fpfit’ command in Stata) without adjustment for other variables. We used STATA version 17.0 for all statistical analysis^(29)^.

### Ethical standards disclosure

This study was conducted according to the guidelines laid down in the Declaration of Helsinki and all procedures involving research study participants were approved by the Norwegian Centre for Research Data (NSD). Participants do not have a unique identification number as data collection is done anonymously and does not contain any sensitive information. Therefore, no additional ethical approval was needed. Participants and their parents were informed that participation is voluntary, and parents had the ability to withdraw their children (<18 years of age) from participation. The present study was however additionally approved by the Data Protection Office at Innlandet Hospital Trust with the reference number 18778329.

## Results

A total of 15 913 adolescents, 57⋅9 % male and 38⋅8 % female, responded to the questionnaire about habits, attitudes and experiences regarding ED consumption. There was an even distribution between the different grades, but with the lowest response rate in grade 13. Most of the participants lived ‘very central’ (69⋅1 %), and 25⋅5 % spent 3–4 h outside school on leisure screen time activities, but the majority were physically active 2–5 times a week (82⋅5 %). The baseline characteristics of the participants are displayed in Table 1.

**Table 1.** Background characteristics

	Total, *n* 15 913 (%)
Sex	
Male	9220 (57⋅9)
Female	6180 (38⋅8)
Missing	513 (3⋅2)
Class	
8th grade	2709 (17⋅0)
9th grade	3260 (20⋅5)
10th grade	3573 (22⋅5)
11th grade	2582 (16⋅2)
12th grade	2135 (13⋅4)
13th grade	1429 (8⋅9)
Missing	225 (1⋅4)
Perceived family economy	
Good	5652 (35⋅5)
Mostly good	5545 (34⋅5)
Neither good or bad	3321 (20⋅9)
Mostly bad	875 (5⋅5)
Bad	265 (1⋅7)
Missing	255 (1⋅6)
Centrality	
Least central	1872 (11⋅8)
Less central	433 (2⋅7)
A bit central	2606 (16⋅4)
Very central	11 002 (69⋅1)
Leisure screen time	
Less than 2 h	2549 (16⋅0)
2–3 h	3390 (21⋅3)
3–4 h	4058 (25⋅5)
4–6 h	3212 (20⋅2)
More than 6 h	2561 (16⋅1)
Missing	143 (0⋅9)
Physical activity	
Never	311 (2⋅0)
Seldom to 2 times a week	2278 (14⋅3)
2–5 times a week	13 124 (82⋅5)
Missing	200 (1⋅3)

**Fig. 1.**
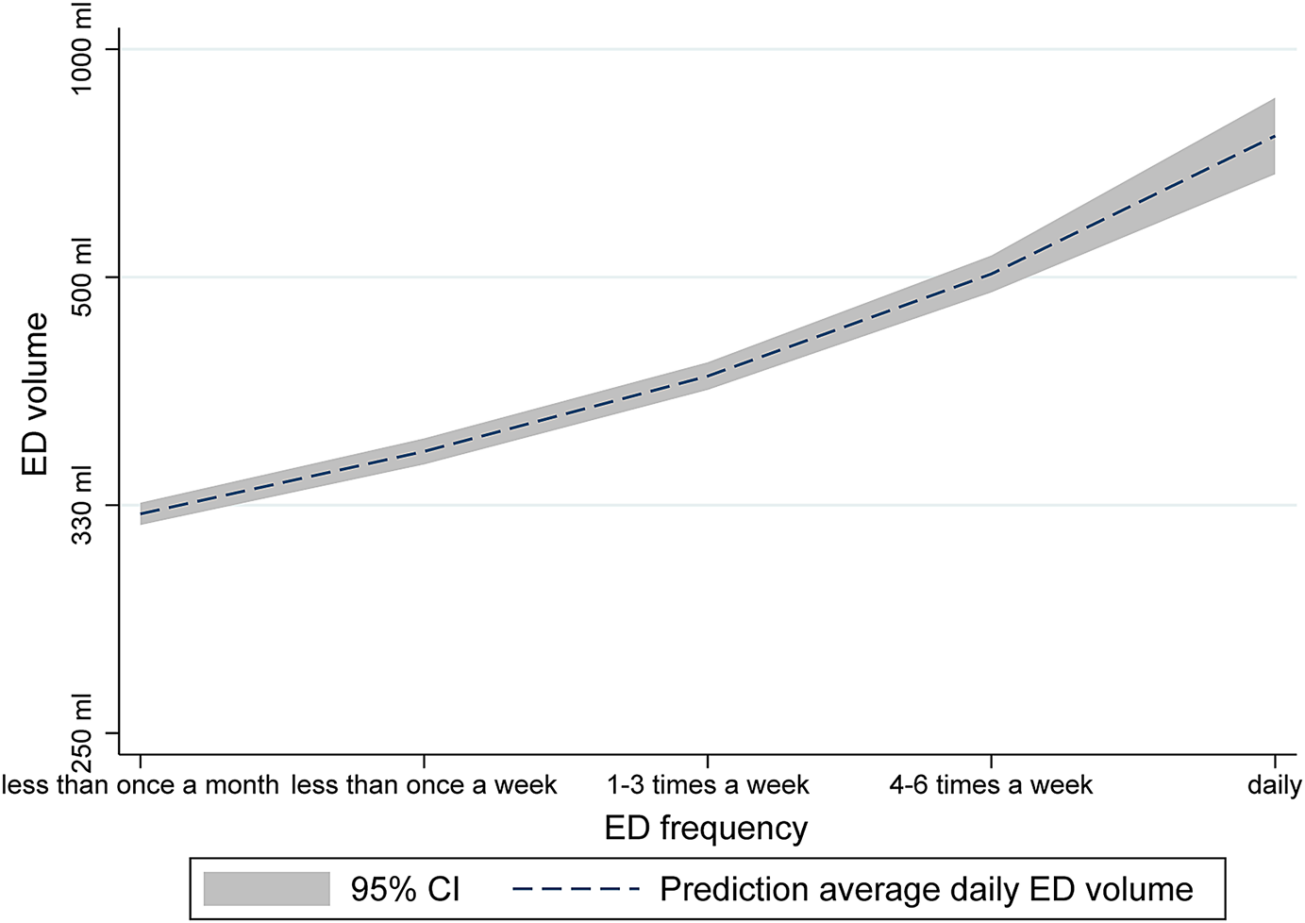
Average daily intake of ED according to ED consumption frequency.

There was a dose-response relationship between the average daily intake of ED and the frequency of ED consumption as shown in Fig. 1. Most responded that they consumed ED ‘because it tastes good’ (96⋅1 %), but also ‘because I need energy’ (47⋅1 %), ‘to stay awake’ (48⋅1 %), ‘to concentrate’ (20⋅4 %) and ‘to perform better in school’ (11⋅3 %) (Table 2). As an example, those who consumed ED to ‘perform better in school’ had an average intake of 193⋅8 ml daily which is 112⋅0 ml (adjusted) more than those who did not report to consume ED for this reason.

**Table 2.** Reasons for consuming ED

	Total, *n* (%)	Mean intake (sd)	Mean difference	Adjusted mean difference
Because it tastes good				
Disagree	605 (3⋅9)	56⋅2 (184⋅0)	Ref	Ref
Agree	14 979 (96⋅1)	78⋅2 (185⋅4)	22⋅0 (6⋅9, 37⋅0)	14⋅2 (−0⋅69, 29⋅1)
Because I need energy				
Disagree	8034 (52⋅9)	61⋅9 (144⋅8)	Ref	Ref
Agree	7159 (47⋅1)	95⋅1 (222⋅7)	33⋅3 (27⋅4, 39⋅2)	31⋅0 (25⋅2, 36⋅9)
Because I need to stay awake				
Disagree	7897 (51⋅9)	55⋅8 (133⋅7)	Ref	Ref
Agree	7317 (48⋅1)	100⋅2 (226⋅1)	44⋅4 (38⋅6, 50⋅3)	34⋅7 (28⋅9, 40⋅6)
Because my friends do it				
Disagree	13 359 (88⋅8)	71⋅1 (160⋅5)		
Agree	1689 (11⋅2)	127⋅7 (319⋅5)	56⋅6 (47⋅2, 65⋅9)	48⋅5 (39⋅2, 57⋅8)
To perform better in sports				
Disagree	13 140 (87⋅3)	67⋅7 (157⋅3)	Ref	Ref
Agree	1903 (12⋅7)	145⋅1 (314⋅4)	77⋅4 (68⋅5, 86⋅3)	69⋅4 (60⋅6, 78⋅3)
To concentrate				
Disagree	11 982 (79⋅6)	59⋅4 (139⋅3)	Ref	Ref
Agree	3072 (20⋅4)	148⋅8 (297⋅7)	89⋅4 (82⋅2, 96⋅7)	73⋅1 (65⋅8, 80⋅3)
To perform better in school				
Disagree	13 302 (88⋅7)	62⋅9 (145⋅6)	Ref	Ref
Agree	1688 (11⋅3)	193⋅8 (358⋅7)	130⋅9 (121⋅7, 140⋅2)	112⋅0 (102⋅7, 121⋅2)

As seen in Table 3, most ED consumers reported that their parents think it is OK that they consume ED (78⋅1 %). Yet, more than 40 % of them responded that their parents had talked to them about ED and that their parents say that they should not drink ED. Approximately 17 % of the adolescents reported that their parents do not know that they drink ED.

**Table 3.** Parental attitudes towards ED consumption

	Total, *n* (%)	Mean intake (sd)	Mean difference	Adjusted mean difference
My parents think it is OK that I drink energy drinks				
Disagree	3356 (21⋅9)	65⋅8 (170⋅3)	Ref	Ref
Agree	11 972 (78⋅1)	80⋅3 (188⋅7)	14⋅5 (7⋅4, 21⋅6)	10⋅2 (3⋅1, 17⋅3)
My parents do not know that I drink energy drinks				
Disagree	12 567 (83⋅0)	74⋅1 (171⋅7)	Ref	Ref
Agree	2569 (17⋅0)	88⋅6 (231⋅3)	14⋅5 (6⋅7, 14⋅3)	11⋅1 (3⋅3, 18⋅8)
My parents say that I shouldn't drink energy drinks				
Disagree	8936 (59⋅1)	73⋅3 (169⋅1)	Ref	Ref
Agree	6171 (40⋅9)	81⋅7 (202⋅5)	8⋅4 (2⋅5, 14⋅4)	6⋅4 (0⋅5, 12⋅3)
My parents have talked to me about energy drinks				
Disagree	7907 (52⋅4)	75⋅0 (176⋅6)	Ref	Ref
Agree	7188 (47⋅6)	78⋅2 (189⋅8)	3⋅2 (−2⋅6, 9⋅0)	2⋅7 (−3⋅1, 8⋅6)

The majority of ED consumers drink ED when they are with friends (59⋅9 %), are at home (54⋅4 %), are gaming (37⋅2 %), or are engaged in other screen-based leisure activities (36⋅2 %) (Table 4). The least reported circumstances were when they are at a youth club (11⋅7 %) or when they are doing sports (16⋅4 %).

**Table 4.** Circumstances when drinking ED

	Total, *n* (%)	Mean intake (sd)	Mean difference	Adjusted mean difference
I am with friends				
Disagree	6054 (40⋅1)	68⋅9 (172⋅7)	Ref	Ref
Agree	9058 (59⋅9)	81⋅7 (189⋅4)	12⋅8 (6⋅8, 18⋅7)	14⋅5 (8⋅5, 20⋅5)
I am at a party				
Disagree	11 130 (74⋅3)	67⋅4 (158⋅1)	Ref	Ref
Agree	3851 (25⋅7)	102⋅3 (238⋅1)	34⋅9 (28⋅2, 41⋅5)	39⋅9 (32⋅9, 46⋅9)
I am on my way to/doing sports				
Disagree	12 509 (83⋅6)	67⋅7 (160⋅2)	Ref	Ref
Agree	2446 (16⋅4)	119⋅3 (260⋅8)	51⋅6 (43⋅8, 59⋅4)	55⋅4 (47⋅5, 63⋅2)
I am at the youth club				
Disagree	13 168 (88⋅3)	64⋅5 (151⋅3)	Ref	Ref
Agree	1743 (11⋅7)	166⋅7 (321⋅2)	102⋅2 (93⋅3, 111⋅2)	101⋅6 (92⋅4, 110⋅8)
I am on my way to or from school				
Disagree	12 574 (84⋅4)	58⋅9 (138⋅4)	Ref	Ref
Agree	2325 (15⋅6)	171⋅0 (315⋅7)	112⋅1 (104⋅2, 120⋅0)	99⋅1 (91⋅1, 107⋅0)
I am gaming/using my computer				
Disagree	9405 (62⋅8)	46⋅0 (124⋅0)	Ref	Ref
Agree	5564 (37⋅2)	128⋅1 (244⋅0)	82⋅1 (76⋅1, 88⋅0)	59⋅8 (53⋅0, 66⋅6)
I am watching TV or movies				
Disagree	9525 (63⋅8)	51⋅5 (135⋅6)	Ref	Ref
Agree	5408 (36⋅2)	120⋅5 (238⋅1)	69⋅0 (63⋅0, 75⋅0)	56⋅2 (50⋅1, 62⋅3)
I am at home				
Disagree	6839 (45⋅6)	45⋅6 (126⋅2)	Ref	Ref
Agree	8165 (54⋅4)	102⋅2 (214⋅7)	56⋅7 (50⋅9, 62⋅5)	45⋅1 (39⋅2, 51⋅0)

Among both ED consumers and high ED consumers, the majority responded that when drinking ED, they ‘do not feel anything in particular’ (65⋅9 %) (Table 5). However, 47⋅2 % ‘felt less tired’ after consuming ED and 33⋅9 % ‘felt stronger’. Adverse effects of ED consumption were in general infrequently reported, with the most common reported side effects being palpitations and sleeping difficulty among more than 10 % of the ED consumers.

**Table 5.** Experiences with ED consumption

	Total, *n* (%)	Mean intake (sd)	Mean difference	Adjusted mean difference
Increased endurance/less tired				
Disagree	7929 (52⋅8)	67⋅4 (167⋅6)	Ref	Ref
Agree	7086 (47⋅2)	87⋅8 (200⋅8)	20⋅4 (14⋅5, 26.,3)	19⋅1 (13⋅2, 24⋅9)
Feel stronger/have extra energy				
Disagree	9870 (66⋅1)	64⋅5 (154⋅0)	Ref	Ref
Agree	5048 (33⋅9)	101⋅5 (230⋅7)	37⋅0 (30⋅7, 43⋅2)	36⋅3 (30⋅2, 42⋅5)
Feel more attractive or popular among friends				
Disagree	13 612 (91⋅5)	69⋅4 (161⋅4)	Ref	Ref
Agree	1259 (8⋅5)	156⋅2 (333⋅5)	86⋅8 (76⋅2, 97⋅3)	80⋅4 (69⋅8, 91⋅0)
Get palpitations				
Disagree	13 449 (90⋅5)	68⋅0 (158⋅4)	Ref	Ref
Agree	1419 (9⋅5)	161⋅0 (329⋅5)	93⋅0 (83⋅1, 103⋅0)	82⋅8 (72⋅9, 92⋅8)
Feel unrest or anxiety				
Disagree	13 581 (91⋅5)	70⋅7 (164⋅2)	Ref	Ref
Agree	1267 (8⋅5)	135⋅7 (305⋅2)	65⋅0 (54⋅6, 75⋅4)	58⋅1 (47⋅6, 68⋅5)
Feel nervous				
Disagree	14 037 (94⋅7)	72⋅1 (168⋅2)	Ref	Ref
Agree	786 (5⋅3)	159⋅1 (347⋅3)	87⋅0 (73⋅9, 100⋅1)	82⋅0 (68⋅9, 95⋅2)
Have difficulty sleeping				
Disagree	12 309 (83⋅4)	72⋅3 (170⋅0)	Ref	Ref
Agree	2446 (16⋅6)	99⋅1 (239⋅2)	26⋅8 (18⋅9, 34⋅8)	25⋅3 (17⋅4, 33⋅1)
Do not feel anything in particular when I drink energy drinks				
Disagree	5059 (34⋅1)	83⋅4 (198⋅4)	Ref	Ref
Agree	9797 (65⋅9)	72⋅8 (175⋅0)	−10⋅6 (−16⋅8, −4⋅4)	−6⋅7 (−12⋅8, −0⋅5)

## Discussion

In this cross-sectional study on attitudes, habits and experiences with ED consumption among Norwegian adolescent, we found that the main reason for consuming ED was the taste, but also the need for energy to stay awake. Many of the adolescents felt more energised after consuming ED and most ED consumers were with friends when consuming ED.

Improved performance is a known marketing strategy for ED producers^(30)^. Indeed, in our study, many adolescents reported that they felt stronger and had more energy after consuming ED. Other studies have also found that adolescents, particularly boys, use ED to improve their performance^(31–33)^ and that adolescents who are physically active drink more ED. Yet, there are also studies that have demonstrated that adolescents who are very physically active are not typical ED consumers^(34)^.

Two-thirds of the adolescents in our study reported that they do not feel anything in particular when drinking ED. This is reflected by the infrequent reporting of side effects such as palpitations, anxiety, nervousness and sleeping difficulties. Moreover, our finding of the majority of participants reporting to drink ED mainly because it tastes good might suggest that there is still limited knowledge of the effects of ED consumption, or that they do not care about the content and possible consequences of frequent ED consumption. Indeed, previous studies have demonstrated that adolescents who consume ED have limited knowledge of the possible consequences of ED and are therefore more likely to consume ED^(5,32)^.

Parental attitudes towards ED consumption can affect adolescents’ habits regarding ED consumption in both directions, influencing them to consume or not. Previous studies have showed that parents play an important role regarding ED consumption where they spark the idea of ED consumption by talking to the adolescents about ED^(32)^. In the present study, many adolescents reported that their parents had talked to them about ED consumption, and several of the adolescents not consuming ED reported that they did not drink ED because their parents did not allow it. Interestingly, neither talking about nor reporting trying to refuse ED consumption was associated with ED intake. Furthermore, most adolescent ED consumers reported that their parents think it is OK that they drink ED. The adolescent period has been demonstrated to be an important period of influence for later food consumption and habits^(35,36)^. Therefore, potential interventions regarding ED consumption might ultimately be most influential in this period.

We found that most ED consumers where either with friends, gaming or doing other screen-based leisure activities when consuming ED. This is in line with previous findings where a social situation, such as being with friends at home or outside, was a common context of ED consumption^(5)^. One could speculate that ED consumption increases among adolescents because of the known exposure to digital marketing of ED^(30)^, through gaming and social media. Moreover, because of these social situations and exposures, ED might have become a common drink to consume just like any other non-alcoholic beverage.

### Strengths and limitations

The main strength of the present study is the large and wide-reaching sample of adolescents across Norway. However, a limitation to the study is the fact that we could not include open questions regarding the reasons for ED use. If the inclusion of such questions had been possible, we could have captured aspects of ED use that our structured questionnaire missed. Such information could also have been obtained using a qualitative or mixed design. Other limitations of the study are that the data are self-reported and are therefore subject to recall-bias.

## Conclusion

The study adds new information on experiences with ED, attitudes towards ED and habits around consumption of ED in adolescents. The experiences and habits regarding adolescent ED consumption are complex and to a certain degree contradictive, yet many adolescents still choose to consume ED in relatively large amounts according to our findings. This information is valuable for strategy and policymakers, next to healthcare professionals and others working with adolescents, when addressing the potential negative affects which comes with ED consumption towards adolescents.
